# A sputum bioassay for airway eosinophilia using an eosinophil peroxidase aptamer

**DOI:** 10.1038/s41598-022-26949-7

**Published:** 2022-12-28

**Authors:** M. Monsur Ali, Michael G. Wolfe, Manali Mukherjee, Katherine Radford, Zil Patel, Dawn White, Julijana Milojevic, Alfredo Capretta, Parameswaran Nair, John D. Brennan

**Affiliations:** 1 Biointerfaces Institute, McMaster University, 1280 Main Street West, Hamilton, ON L8S 4O3 Canada; 2 Division of Respirology, McMaster University, and Firestone Institute of Respiratory Health at St. Joseph’s Health Care, Hamilton, ON L8N 4A6 Canada

**Keywords:** Asthma, Biochemical assays, Assay systems, DNA

## Abstract

Eosinophils are granulocytes that play a significant role in the pathogenesis of asthma and other airway diseases. Directing patient treatment based on the level of eosinophilia has been shown to be extremely effective in reducing exacerbations and therefore has tremendous potential as a routine clinical test. Herein, we describe the in vitro selection and optimization of DNA aptamers that bind to eosinophil peroxidase (EPX), a protein biomarker unique to eosinophils. Fifteen rounds of magnetic bead aptamer selection were performed prior to high throughput DNA sequencing. The top 10 aptamer candidates were assessed for EPX binding using a mobility shift assay. This process identified a lead aptamer candidate termed EAP1-05 with low nanomolar affinity and high specificity for EPX over other common sputum proteins. This aptamer sequence was further optimized through truncation and used to develop an easy-to-use colourimetric pull-down assay that can detect EPX over a concentration range from 1 – 100 nM in processed sputum. Forty-six clinical samples were processed using a new sputum dispersal method, appropriate for a rapid assessment assay, that avoids centrifugation and lengthy processing times. The assay showed 89% sensitivity and 96% specificity to detect eosinophilia (compared to gold standard sputum cytometry), with results being produced in under an hour. This assay could allow for an easy assessment of eosinophil activity in the airway to guide anti-inflammatory therapy for several airway diseases.

## Introduction

With a worldwide prevalence in over 300 million people, asthma remains as one of the most widespread chronic diseases^1^. In Canada, this translates into a ~ $2.1 billion dollar healthcare burden per annum^2^. One limitation to current clinical asthma management is correctly and rapidly identifying the underlying causes of bronchial inflammation, and personalizing treatment based on those findings. The current gold standard for patient assessment involves sputum induction followed by histological staining, which has been proven to be extremely effective in managing asthma, and other airway diseases such as chronic cough and chronic bronchitis^3–5^. However, this sputum cytology technique is limited in application owing to accessibility to a laboratory, and the need for equipment and trained technicians to perform the testing. While testing of blood eosinophils has been proposed as a simpler method for assessing airway inflammation, the correlation and concordance with sputum eosinophil counts is very poor in severe patients who are on high doses of inhaled or oral glucocorticosteroids^6^. Further, blood eosinophils do not reflect eosinophilic exacerbations of patients with severe asthma who are being treated with anti-eosinophilic biologics^7^, thus indicating that sputum eosinophil counts are the gold standard to assess airway luminal eosinophilia.

A simpler alternative to assess sputum eosinophilia is to perform high-throughput assays such as an enzyme-linked immunosorbent assay (ELISA) on the fluid-fraction of the processed sputa, using surrogate markers for eosinophils, a leucocyte that is highly linked to prevalence and severity in asthma^4,8–10^. However, this approach is also limited by the need for lengthy processing times, multiple assay steps to separate cellular and fluid fractions, and the need for expensive instrumentation. Hence, there remains a major need for simple diagnostic platforms to rapidly identify and quantify the presence of eosinophils in sputum samples.

The use of eosinophil peroxidase (EPX) has been validated as an ideal and specific biomarker to identify the presence of eosinophils when compared to other surrogate markers^11^. While antibodies have already been developed for this target, recent research has shown that the possibility of an autoimmune response to eosinophil degranulation products such as EPX can result in a high polyclonal IgG presence in the airways^11^. This high IgG presence can complicate rapid antibody lateral flow detection systems, requiring additional sample processing steps^12^. As an alternative, DNA aptamers have garnered substantial interest as biorecognition elements. Since their discovery in the early 1990’s, aptamers have been growing in popularity due to their low cost, high stability and ease of chemical modifications^13,14^. Using systematic evolution of ligands by exponential enrichment (SELEX), aptamers can be identified for a variety of targets^15^. To the best of our knowledge, no nucleic acid aptamer has been reported for EPX. Given the clinical value of EPX as a diagnostic marker, anti-EPX aptamers have the potential to greatly improve detection platforms for identifying eosinophils to aid in diagnosis of airway diseases.

Herein, we report on the selection and optimization of a new DNA aptamer for EPX, and its use for the development of an agarose bead-based colorimetric pull-down assay to detect EPX in patient sputum samples. A rapid sputum processing method is also reported that reduces both the time and technical complexity of sample preparation, resulting in a simplified assay that can be performed in under 1 h. The validation of the pull-down assay using clinical samples from both eosinophilic and non-eosinophil sputum (sputum eosinophils > 3% or < 3%)^16^, as determined by routine sputum cytology and ELISA assays, is also reported, showing excellent clinical selectivity and specificity for the new assay with no interferences from autoantibodies.

## Results and discussion

**Figure 1:**
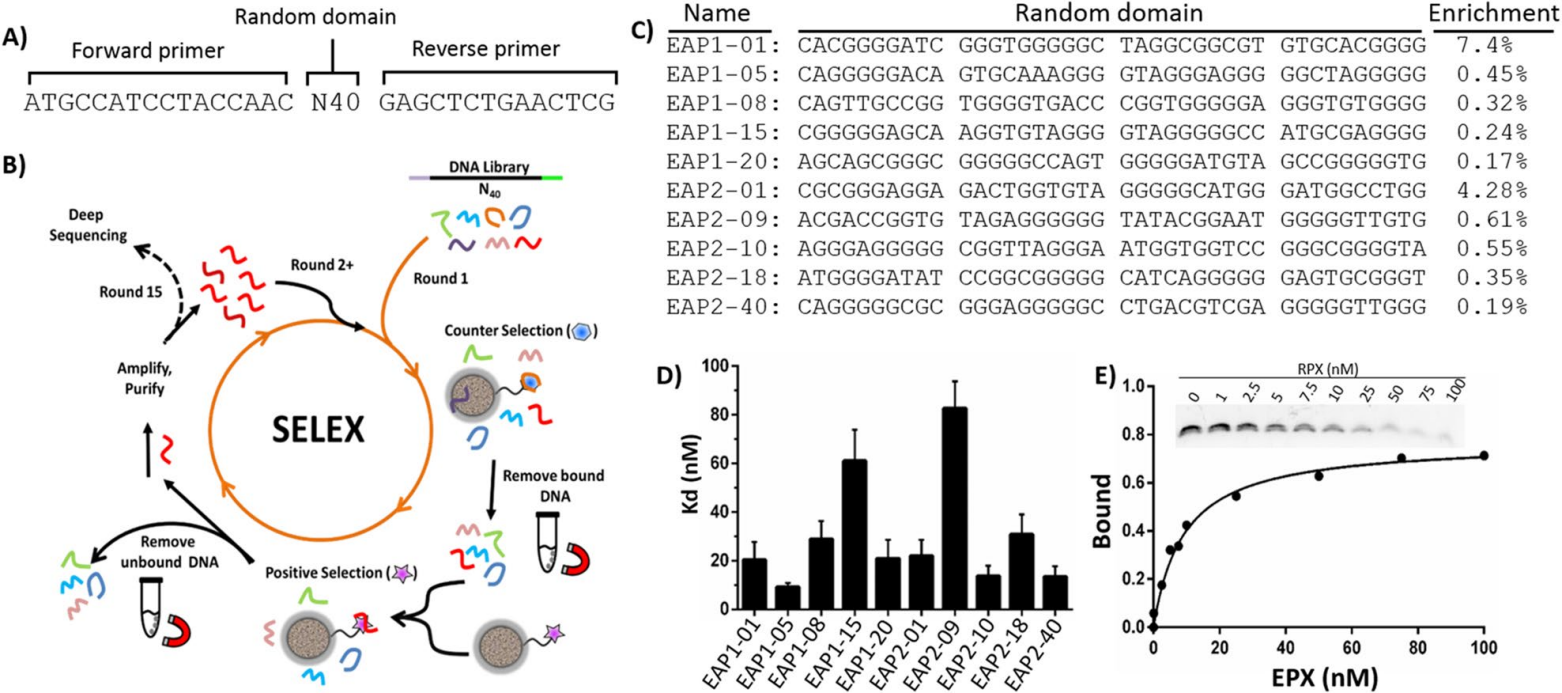
Selection of EPX-binding DNA Aptamer. (**A**) DNA library used in the selection. N40 in the DNA library represents 40 random nucleotides in the middle of the library and the fixed arms on each side represent the primer binding regions for PCR amplification. (**B**) Schematic illustration of the in vitro selection method (see details in the experimental section). (**C**) Top 5 sequences from each type of selection including the percent of enrichment during selection. (**D**) Dissociation constants (K_d_) determined from electrophoretic mobility shift assays (EMSA) for each of the aptamer sequences shown in C. (**E**) Binding isotherm for aptamer EAP1-05 determined from EMSA experiments.

To identify DNA aptamers for EPX, SELEX experiments were carried out using magnetic beads (MB) with immobilized EPX and a DNA library (DNA_L_) containing 40 random nucleotides flanked by fixed domains in each end to serve as PCR primer binding arms (Fig. 1A, See Table S1 for the sequences of all oligonucleotides used in this work). The protocol used for the selection experiments is shown in Fig. 1B, and a detailed procedure of the selection experiment is provided in the experimental section. Briefly, either EPX (for positive selection) or myeloperoxidase (MPO, for negative selection) were covalently conjugated to N-hydroxysuccinimide-activated magnetic beads following the manufacturer’s instructions. The selection process began with a negative selection wherein the DNA_L_ was incubated with the MPO-coated MBs. After incubation, the MBs were magnetically separated and the unbound free DNA sequences in the supernatant were collected, amplified by PCR to the reach the original concentration of the library, and employed in the positive selection with EPX-coated beads. In the positive selection, the unbound free sequences were discarded. After washing the beads, the bound DNA molecules were dissociated from the bead, isolated, and amplified by PCR. The sense strands of the PCR products were then purified by denaturing polyacrylamide gel electrophoresis (dPAGE) and employed in the next round of negative selection. Since EPX is a highly cationic protein, the selection was conducted in a buffer with a high pH (9.0) and high salt concentration (1.0 M NaCl) to reduce the non-specific electrostatic interactions between EPX and DNA_L_. To further improve the specific binding of the DNA pool with EPX, the PCR product of round seven was divided into two pools called EAP1 and EAP2. The initial magnetic bead selection strategy was continued with the EAP1 pool, and a parallel selection was carried out with the EAP2 pool where a hairpin DNA (HPD) was added to the MB-EPX suspension prior to adding the DNA pool. It was anticipated that the HPD should serve as a blocker to bind to the EPX and beads. Thus, only the high affinity and specific DNA molecules in the library should be able to bind to EPX.

After 15 rounds of selection, the DNA molecules bound to the EPX-coated beads from each selection were eluted by adding free EPX instead of heating to ensure that the DNA molecules bound to free EPX. Eluted sequences were PCR amplified and the pool was used for deep sequencing. The top 5 sequences of each selection are shown in Fig. 1C. The sequencing results revealed that most of the sequences were guanine rich, suggesting the presence of a G-quadruplex structure^17,18^. The binding affinity of these aptamer candidates was evaluated by electrophoretic mobility shift assays (EMSA) using fluorescently labeled aptamers (Fig. 1D,E). It was surprising to note that the bands of aptamer-EPX complexes did not appear in the gel while the free aptamer band intensities diminished. We found that when the aptamer formed a complex with EPX the fluorescence of the bound aptamer was strongly quenched (see below). Therefore, the binding affinity of each aptamer was calculated based on the reduction of the band intensity of the free aptamer in the gels. High affinity aptamers were identified in each of the EAP1 and EAP2 pools, however, aptamer EAP1-05 was found to have the highest binding affinity with a *K*_*d*_ of 9.2 ± 1.6 nM (Fig. 1E; note that the unprocessed gel image of Fig. 1E is shown in Fig. S1). These results suggest that the addition of the hairpin DNA was not required to eliminate non-specifically bound DNA sequences, as both pools showed similar affinity trends.

**Figure 2:**
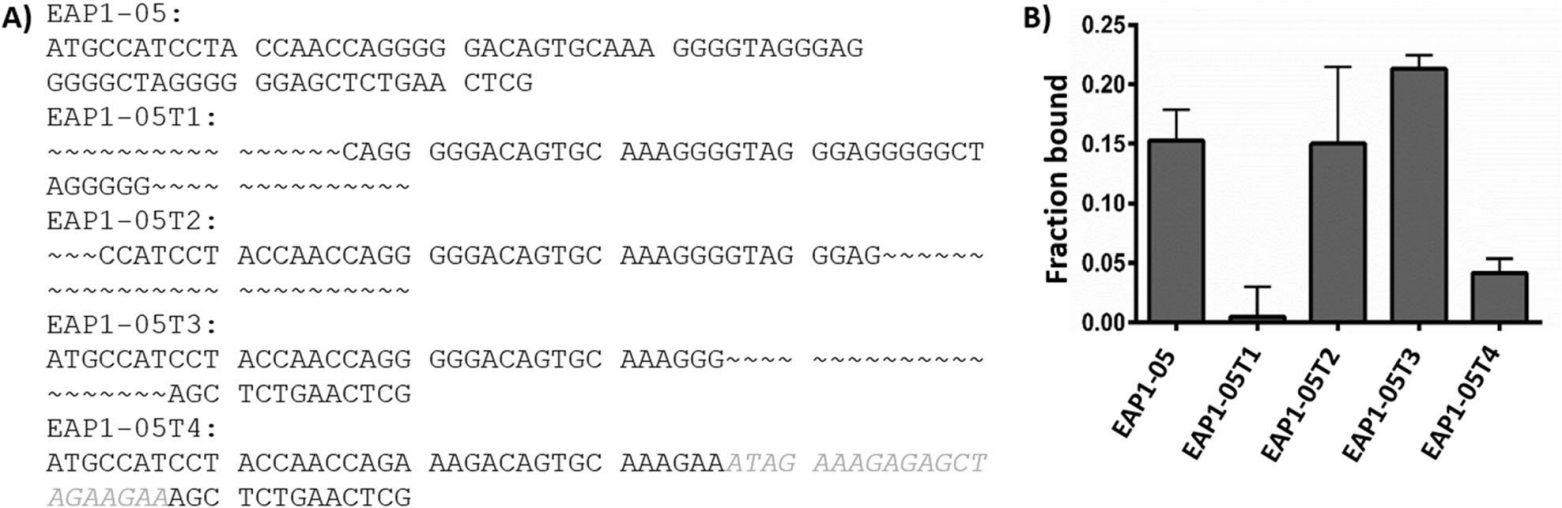
Truncation study of EAP1-05 and binding assay. (**A**) Full length and truncated sequences. Curved dashes denote deleted bases. Light grey bases in EAP1-05T4 represent replaced bases. (**B**) Single point EMSA-based binding assays of the sequences shown in (**A**) using 3 nM aptamer and 5 nM EPX.

Next, EAP1-05 was subjected to truncation and deletion experiments to shorten the aptamer and optimize its binding ability. The aptamer was truncated by removing various DNA segments (Fig. 2A), and the fraction of aptamer bound to EPX was assessed by EMSA using 3 nM of aptamer and a single EPX concentration of 5 nM and calculating the decrease in free aptamer fluorescence in EMSA gels. The results showed that removing the 3’ stem loop of EAP1-05 significantly reduced the fraction of bound aptamer (EAP1-05T1, Fig. 2B), while deletions from the middle of the sequence had a small positive impact on EPX binding capabilities (particularly for EAP1-05T3). Interestingly, switching several guanosine residues for adenosine residues also resulted in a significant reduction in EPX binding (Fig. 2, EAP1-05T4), further supporting the hypothesis that the aptamer likely contained a G-quadruplex motif. Overall, the EAP1-05T3 aptamer showed the highest degree of EPX binding, and thus was further characterized. The predicted secondary structures of EAP1-05 and EAP1-05T3 are shown in Fig. S2 in the ESI^19,20^.

Initial attempts to assess the binding affinity of the aptamer using solution-based fluorescence intensity assays demonstrated that the binding of EPX led to substantial quenching for both the native and truncated versions of the EAP1-05 aptamer (up to 70% quenching at 75 nM EPX, see Fig. S3 in the ESI), with minimal quenching from non-target proteins such as MPO, confirming that quenching of fluorescence was responsible for the lack of an EMSA band for the aptamer-EPX complex. It is well known that the heme group within proteins can produce substantial quenching of fluorophores, including fluorescein, when it is within 10 nm of the fluorophore, owing to non-radiative energy transfer^21^. This supports the binding of EPX to the aptamer and quenching of the FAM label by the heme group in EPX. The lack of quenching by MPO and other peroxidases (which also contain heme groups) suggest that these do not bind to the aptamer.

**Figure 3:**
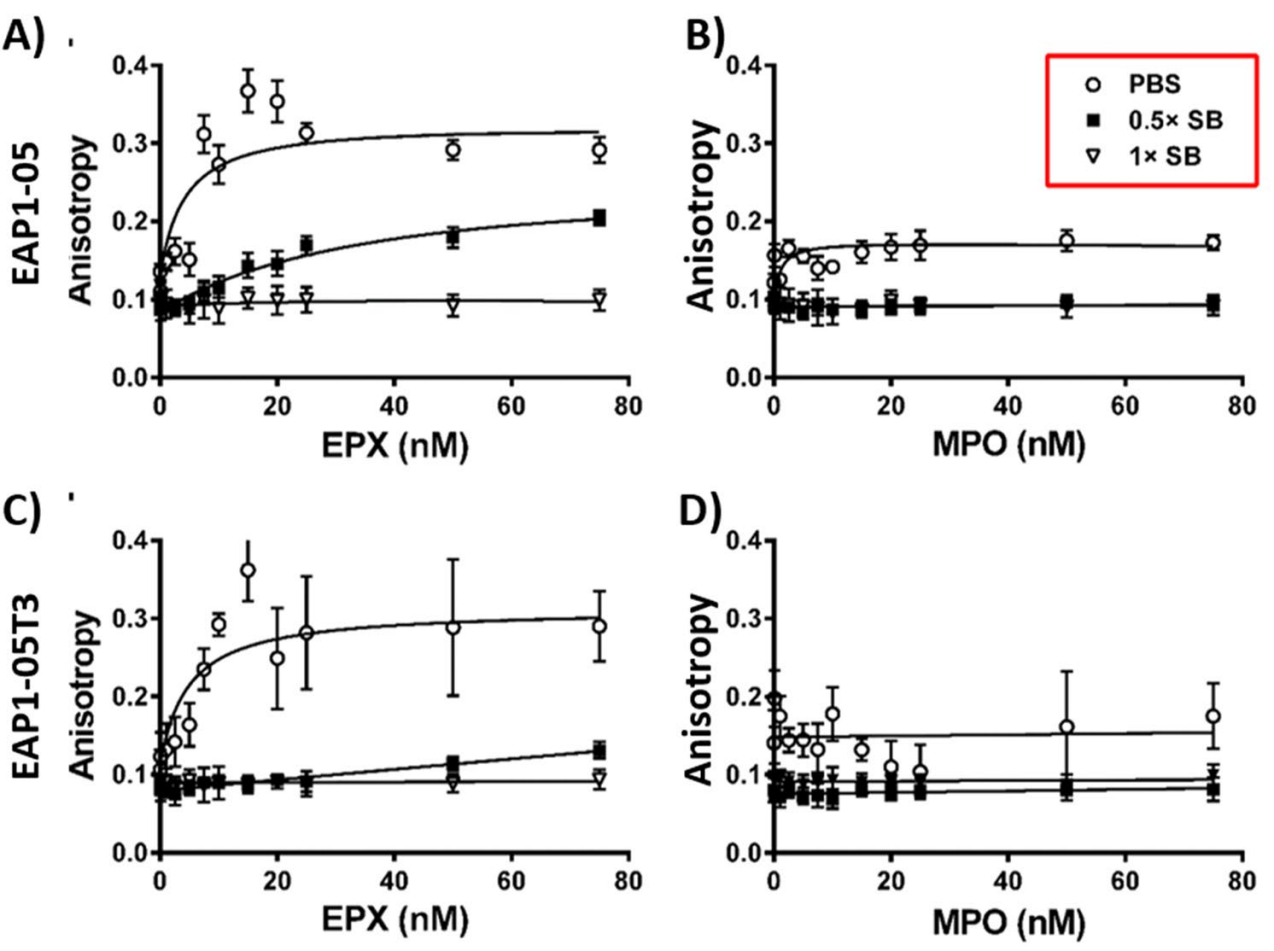
Effect of buffer on binding affinity and specificity of the EAP1-05 and EAP1-05T3 aptamers. (**A**) Fluorescence anisotropy of EAP1-05 aptamer with EPX and (**B**) MPO in PBS (white circles), 0.5 × SB (black squares), and 1 × SB (white triangles). (**C**) Fluorescence anisotropy of EAP1-05T3 aptamer with EPX and (**D**) MPO using the buffers listed in (**A**). Error bars represent the standard deviation of triplicate measurements.

To further assess binding, fluorescence anisotropy assays were also performed with both the full length EAP1-05 and the truncated version EAP1-05T3 to assess the affinity of aptamers for EPX using different buffer conditions. No anisotropy changes were observed for either EAP1-05 or EAP1-05T3 when using the original selection buffer (1 × SB; Fig. 3A,C). Diluting the buffer to 0.5 × SB resulted in an increase in anisotropy upon binding of EPX, with a calculated *K*_*d*_ of 36.6 ± 4.8 nM for the EAP1-05 aptamer (Fig. 3A). We hypothesize that the lower salt concentration of the 0.5 × SB likely results in reduced masking of charges on EPX, increasing the affinity between EPX and EAP1-05. However, it was interesting to observe that the EAP1-05T3 aptamer had very little binding affinity to EPX despite the reduced salt concentration. To further investigate the importance of the binding conditions, the interaction between EPX and EAP1-05 or EAP1-05T3 was investigated in PBS (10 mM Na_2_HPO_4_, 1.8 mM KH_2_PO_4_, 137 mM NaCl, 2.7 mM KCl, pH 7.4). Interestingly, the binding affinity was further improved using the lower ionic strength buffer, with a *K*_*d*_ of 4.3 ± 1.6 nM and 4.8 ± 2.0 nM for EAP1-05 and EAP1-05T3, respectively (Fig. 3A,C). However, the low ionic strength buffer also resulted in slightly increased non-specific binding to MPO for both aptamers (Fig. 3B,D). Since both the full length and the truncated aptamer produced almost identical *K*_*d*_ values, the truncated EAP1-05T3 aptamer was used to develop a colorimetric assay for EPX as the shortened aptamer is easier and less costly to synthesize.

**Figure 4:**
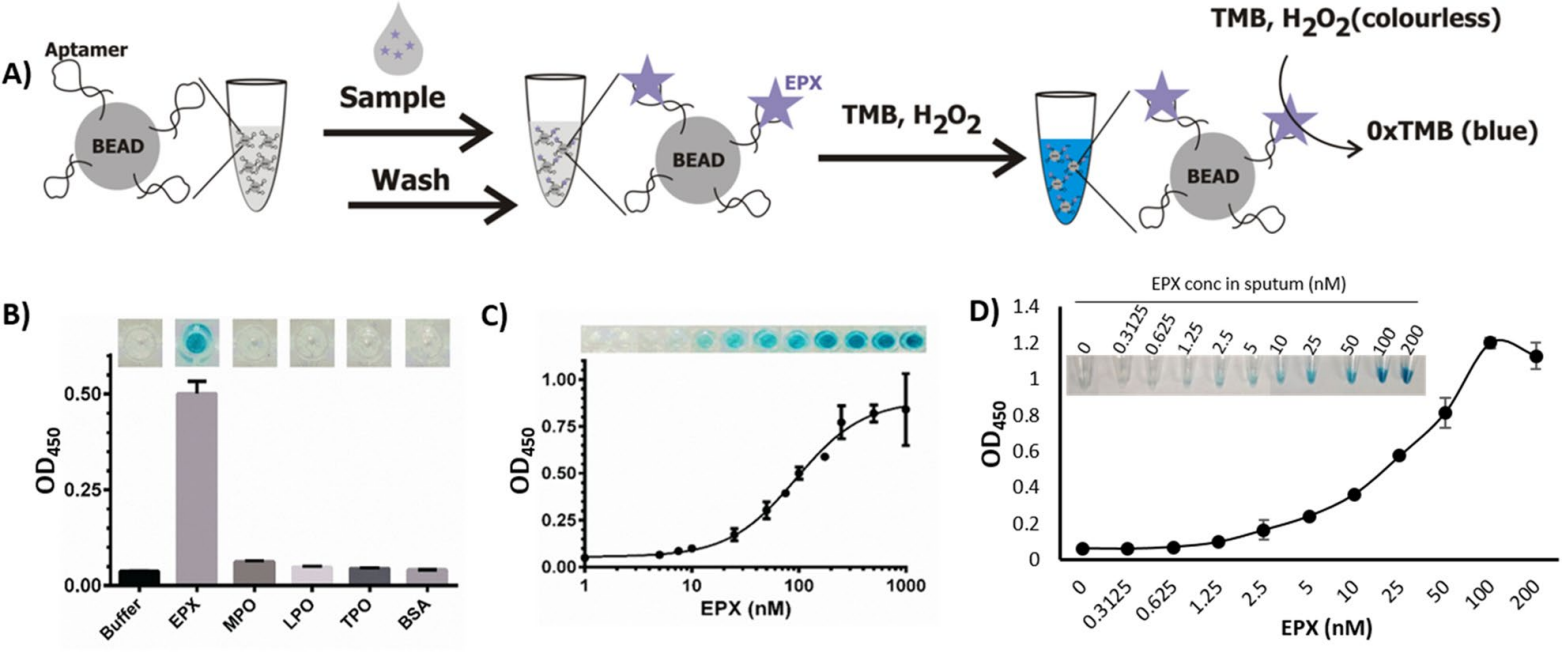
Colorimetric assay for EPX. (**A**) Schematic illustration of the EPX pull-down assay. (**B**) Color images and OD_450_ of quenched samples to evaluate the selectivity of the aptamer-pulldown assay. (**C**) Color images and OD_450_ of quenched samples to evaluate the concentration–response and limit of detection of the pulldown assay using EPX in HEPES buffer. (**D**) Color images and OD_450_ values of quenched samples to evaluate the concentration–response and limit of detection of the pulldown assay using EPX spiked into 25% sputum.

To generate a simple colorimetric assay for EPX, we took advantage of the inherent peroxidase activity of EPX to catalyze hydrogen peroxide-mediated oxidation of 3,3′,5,5′-tetramethylbenzidine (TMB), which produces a blue color that can be easily seen by eye. The conceptual design of the assay is illustrated in Fig. 4A. First, a 5′-biotinylated version of EAP1-05T3 aptamer with a T_10_ extension (see the sequence in Table S1 in the ESI) was immobilized on streptavidin-coated agarose beads, washed to remove unbound aptamer sequences and suspended in the PBS binding buffer. Next, EPX was added and allowed to form a complex with the aptamer on the beads. The unbound EPX molecules in the supernatant were then removed using 3 washing cycles (300 µL PBS per washing cycle) and the beads containing the captured EPX molecules were transferred to a fresh buffer solution. Finally, a standard solution of H_2_O_2_ and TMB^22,23^ was added and allowed to react for 5 min to produce a blue color in the reaction tube, which was imaged with a smart phone camera. For analysis using an absorbance spectrophotometer, the reactions were quenched by adding an equal volume of 0.5 M HCl, producing a yellow product. This reaction mixture was transferred to a microwell plate and the absorbance was measured using a Tecan M200 platereader operating at 450 nm. For visual detection, the blue colour present in Eppendorf tubes was directly observed by eye.

The assay was first optimized using pure EPX with two different buffer conditions: 0.5 × SB and HEPES buffer (HB, see Table S2). The results presented in Fig. S4 showed that HB produced a significantly stronger color, which is consistent with the fluorescence anisotropy results that demonstrated better binding with lower ionic strength buffers. Therefore, HB was used in the TMB colorimetric assay development. Using this new buffer, we evaluated the selectivity of the pulldown assay by comparing the signal produced by EPX (100 nM) to that of several other granulocytic proteins (each at 1 µM). The results provided in Fig. 4B showed that, despite the high similarity between EPX and several similar peroxidase proteins, the assay only produced a signal in the presence of EPX (Fig. 4B). This was encouraging as EPX and MPO share 70% amino acid sequence identity and 81% sequence similarity according to a protein BLAST search (EPX UniProt #P11678, MPO #P05164)^24^. Next, the analytical sensitivity of the assay was determined using pure EPX with concentrations spanning the clinically relevant range of 1 nM–1 µM (77 ng/mL–77 µg/mL)^10,12,25^. This resulted in a limit of detection (LoD) of 1 nM (blank + 3σ) when using a plate reader, although the naked-eye limit of detection was approximately 25 nM (based on the images of the microwell plates shown in Fig. 4C). The LoD was also investigated in spiked sputum (sputum samples were spiked with varying concentrations of EPX and diluted to 25% with assay buffer) and produced a similar LoD of 1.25 nM using a plate reader to measure absorbance (Fig. 4D), indicating that the assay is compatible with complex sputum samples. These LOD values are also comparable to a published antibody-based EPX assay (circa 6 nM EPX)^12^.

To further validate the aptamer-pulldown assay, we next evaluated the assay with patient sputum samples. A total of 46 sputum samples were obtained from patients (n = 36) or healthy donors (n = 10). All experiments with patient samples were performed as per the protocols approved by the Hamilton integrated Research Ethics Board (HiREB), St. Joseph’s Hospital, Hamilton, project number: 12-3687. Healthy donors were recruited under the approved HiREB protocol #13203 and signed written consent was obtained for this clinical validation study. A two-way blinded protocol with samples identified using unique 5-digit random numbers was used, wherein the cytologist obtained clinical cell differentials on the sputum with knowledge of the patient particulars but did not know which samples were sent for aptamer analysis, while the scientist running aptamer assays or ELISA was not aware of the cell differential values.

Patient samples were derived from sputa that were clinically indicated for a cell differential with excess available sample after routine processing was done. Healthy donors were identified as those with no known respiratory disease, infection or symptoms, not within 8 weeks of any vaccination, non-smoking and generally deemed to be in good health. The sputum plugs were split into two equal volumes and the first aliquot was processed using a 4:1 mixture of PBS and 0.1% dithiothreitol (DTT) as per the “gold-standard” clinical method^25^ while the second aliquot was dispersed with HEPES buffer containing 2 mM DTT for use in the pull-down assay (see below). For the gold-standard clinical method, the sputum was first centrifuged and then the suspended cells were smeared onto a slide to produce cytospin slides. The cells were then stained with Wright’s stain, and cellular differentials (eosinophils/neutrophils) were determined by manual counting of eosinophil and neutrophil cells, and reported as a percentage of a total of 400 cells counted by a cytologist (validated for clinical routine use, see Table S4 for cell counts in each of the 46 samples)^26,27^. Matched cell-free supernatants (fluid fraction) were assessed for EPX reactivity by a traditional ELISA method as previously described^26,27^.

Samples were designated as eosinophilic based on the presence of intact eosinophils (≥ 3%) and/or free eosinophil granules. In the event where the eosinophil numbers may have been masked^28^ by high total cell count and neutrophils (in a patient undergoing an infective exacerbation), free eosinophil granules or EPX (ELISA) was used to assess/confirm the underlying eosinophilia. Based on these assays, the samples were stratified as: 1) healthy donor samples, confirmed to have no evidence of inflammation, which were indicated as eosinophil (EOS) negative; 2) patient samples with < 3% eosinophil content and low neutrophil counts (< 64%, sputum total cell count; < 9.7 × 10^6^ cells/g), denoted as EOS negative; 3) patients with mixed granulocytic sputa with total cell count < 9.7 × 10^6^ cells/g, neutrophils > 64% and eosinophils ≥ 3%, or presence of free eosinophil granules, were indicated as EOS positive; and 4) EOS positive samples with ≥ 3% eosinophil levels, and/or free eosinophil granules and low neutrophil counts (< 64%, sputum total cell count < 9.7 × 10^6^ cells/g). The threshold of 9.7 × 10^6^ cells/g is based on the 90th percentile of sputum cell differentials from 120 normal healthy donors^16^, which has been clinically validated and used in clinical practise. Total cell counts greater than 9.7 × 10^6^ cells/g is indicative of an infection, in particular when above 20 million it is an ongoing airway bacterial infection. We note that mixed granulocytic sputa were considered to be EOS positive as they have evidence of eosinophil activity (free granules or EPX assay) since the eosinophil numbers are masked by high neutrophils on a sputum differential count. 28 samples were identified as EOS negatives and 18 were EOS positives which included patients with mixed sputa.

Initial pulldown assays on patient samples used the same DTT/PBS buffer used for the gold-standard cell-counting assay. However, it was determined that this buffer was not compatible with a peroxidase assay, as an initial test of 4 negative and 3 positive samples resulted in none of the samples producing a color (Fig. S5A), even though many of the positive samples contained high amounts of EPX based on the ELISA data (see Fig. 6 below). Further investigation of the sample processing method indicated that the high amount of DTT (0.1%) used in the routine clinical processing interfered with the EPX reaction as the hydrogen peroxide immediately reacted with the DTT (Fig. S5B). A reduction of the DTT to 2 mM in HEPES buffer (HB) produced the required dispersal of the sputum sample while retaining the ability to generate a color from the aptamer-based peroxidase assay (Fig. S5C). The final sample processing method for the pulldown assay involved dispersing the sputum samples 1:8 w/v in HEPES buffer (composition: 50 mM HEPES, 300 mM NaCl, 15 mM MgCl_2_, 0.01% Tween 20, 2 mM DTT). The dispersed plugs were then inverted and shaken for 5 min by hand, and then allowed to settle on ice for 2 min, after which the supernatants were aliquoted and stored at − 20 °C for later analysis. An anti-protease cocktail inhibitor containing a mixture of serine, cysteine and metalloproteases was added to each aliquot of cell-free supernatant to prevent degradation of the EPX target protein, as previous work^29^ has shown slow degradation of fluid-phase analytes in the cell-free supernatants during long term storage. This new sputum processing method reduced both the number of steps and technical complexity of sample processing, reducing processing time from 1 h to 10 min (see Fig. S6).

**Figure 5:**
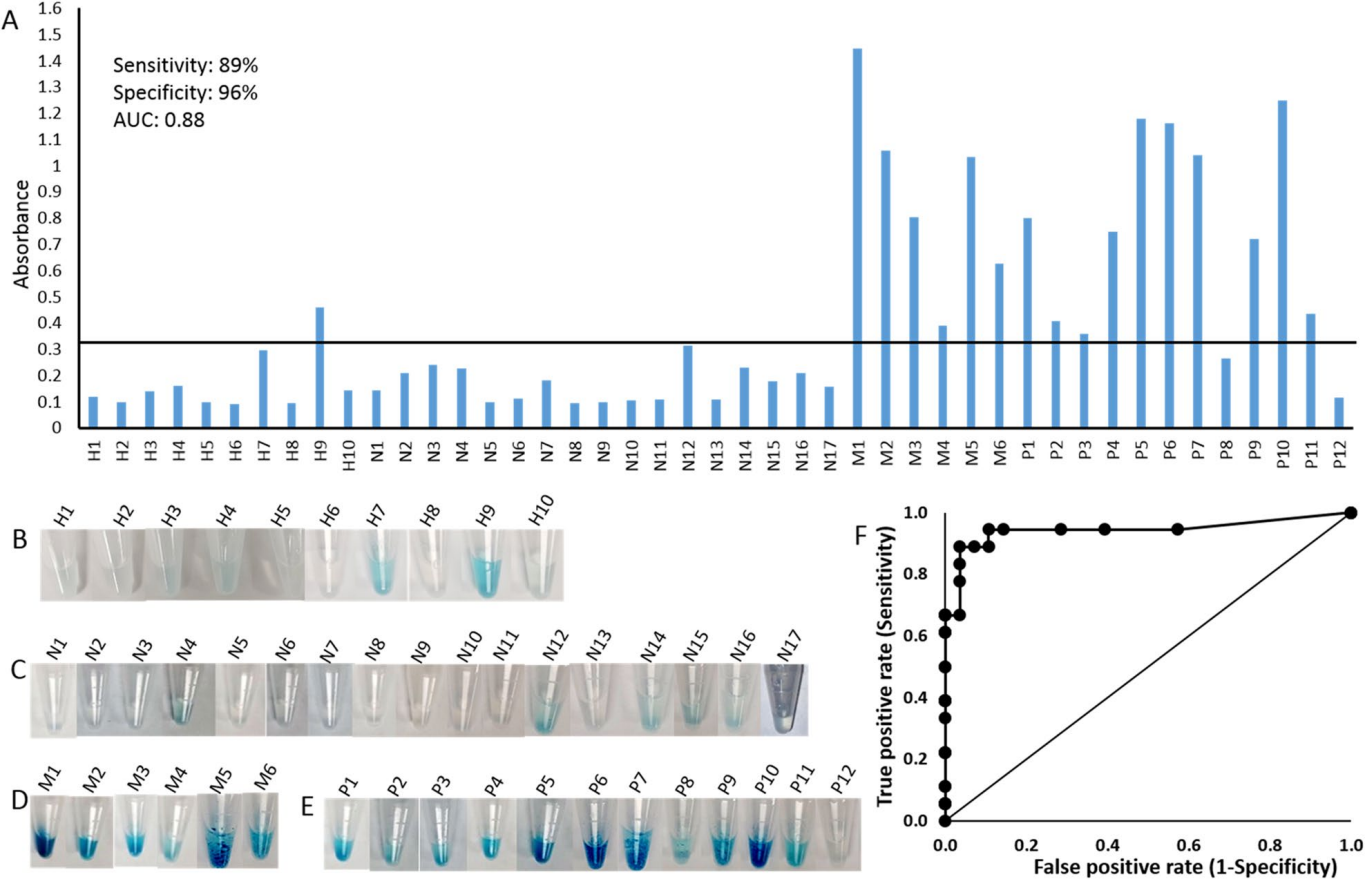
Evaluation of EPX pulldown assay with patients’ sputum samples. (**A**) OD values of EPX pulldown assay for a total of 47 clinical samples. Images of colorimetric outputs for (**B**) healthy (H); (**C**) negative (N); (**D**) mixed (M); and (**E**) positive (P) samples. (**F**) Reciever-operator characteristic plot for absorbance data shown in Panel (A).

Figure 5A shows the comparison between the “gold-standard” assay described above and the aptamer pull-down assay. The aptamer pulldown assay was conducted on the matched “HB buffer dispersed” samples for each of the 46 samples and results were compared with the gold standard. The raw OD values are shown in Fig. 5A, while visual results for the assays are shown in Fig. 5B–E. Visual data for the healthy cohort samples prior to the pull-down assay are shown in Fig. S7 along with the images following the pull-down assay. This data shows that even healthy sputum samples can produce high colorimetric signals (3/10), demonstrating that simple addition of H_2_O_2_/TMB to sputum samples without the pull-down step will lead to an unacceptably high level of false positives owing to the presence of other peroxidases. Following the pull-down step, 2 of the 3 false positives show a marked reduction in color while one sample with a particularly high initial TMB signal showed a substantial reduction in color, but as seen in Fig. 5A is still observed to be a false positive. The data in Fig. 5A is plotted based on inflammatory status (gold standard): healthy (n = 10; negative) and non-eosinophilic (n = 18; negative); vs. mixed-eosinophilic (n = 6; mixed) and eosinophilic (n = 12; positive). Using an OD cut-off of 0.32 based on maximizing the sum of (sensitivity + specificity) on a receiver-operator characteristic (ROC) plot (Fig. 5F), 16/18 samples are identified as true positives (sensitivity of 89%) while 27/28 samples are identified as true negatives (specificity of 96%), with an area under the curve of 0.88.

**Figure 6:**
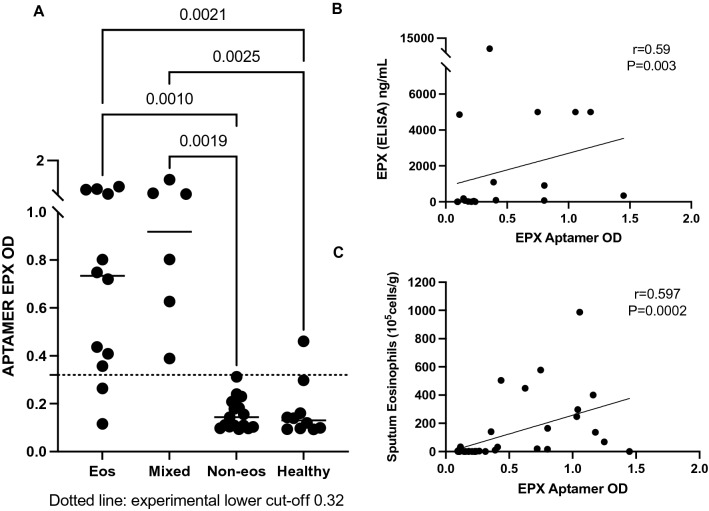
Statistical Analysis of Clinical validation and correlation data. (**A**) Scatter plots showing distribution of aptamer EPX OD data for eosinophilic, mixed granulocytic (evidence of eosinophilia and neutrophils) and non-eosinophilic samples (negative) samples. The cut-off value 0.32 was assessed based on the AUC (Receiver-operating curve) using values generated from n = 18 negative samples, Fig. 5F. Negative samples were confirmed by absence of eosinophils and free eosinophil granules using gold standard (routine sputum cytology) (**B**) Correlation between aptamer assay OD values and percent eosinophils present in sputum samples. (**C**) Correlation between aptamer assay OD values and total eosinophils present in sputum samples. Each symbol is representative one patient/individual value. Kruskal Wallis and Spearman correlation test. P < 0.05 considered as significant.

Additional analysis of the assay data (Fig. 6) was performed to assess the performance of the pulldown assay for positive, mixed, healthy and negative samples.  Fig. 6A demonstrates that the aptamer-based pulldown assay provided OD values for eosinophil samples that were significantly greater than for non-eosinophilic samples (P < 0.003 in all cases), even when using mixed samples. Hence, the current pull-down assay could correctly predict eosinophilic inflammation in those samples where the routine sputum cytology using percentage of eosinophils would have failed (mixed granulocytic samples), since high neutrophil counts can mask eosinophilia in close to a third of patients who may have intense neutrophilia^30^ when using cell differential counts. The measurement of EPX overcomes this disadvantage, as previously demonstrated by conventional ELISA^25^ and further confirmed by the EPX values from the pull-down assays, which correlated positively with the percent eosinophils (Fig. 6B, r  = 0.59, P = 0.003) and absolute eosinophils (Fig. 6C, r = 0.60, P < 0.001) detected in the sputum using the gold-standard clinical method, highlighting the diagnostic utility of the pulldown assay.

Given the accuracy of the aptamer-based pulldown assay for detection of EPX in clinical samples, the assay is expected to be useful in clinical practice to identify airway eosinophilia, to initiate and monitor response to anti-inflammatory treatments such as corticosteroids and anti-eosinophil biologics, and to evaluate new therapies directed against eosinophils. A key advantage of the assay is the ability to detect EPX in eosinophil positive samples even when there are high levels of anti-EPX autoantibodies present, which was not possible using antibody-based lateral flow or ELISA methods. The ELISA method produced low OD values even for positive samples with high aptamer OD values due to autoantibody interference and possible masking of epitopes (see Fig. S8). This shows a key advantage of aptamers in that they can bind to epitopes that are not blocked by autoantibodies. The inherent stability of aptamers also makes them better suited to use in low-income countries with tropical climates and high humidity, where antibodies may be unstable.

Overall, the new assay method provides a simple and accurate method for quickly assessing airway eosinophilia that overcomes the drawbacks of both the current gold-standard cell differential assay and the ELISA and lateral flow assays. As such, it should be much easier to implement the pulldown assay in routine clinical practice without the need for specialized laboratory equipment or technical staff. We have further simplified the sample processing using a dispersal method that requires less than 10 min and no specialised equipment, potentially making the assay suitable for use at the point-of-need to help guide anti-inflammatory treatment (both inhaled and oral corticosteroids and biologics) for a wide range of airway diseases such as asthma, chronic cough, and chronic bronchitis.

The current method provides a clinical sensitivity of 89% and clinical specificity of 96%. However, the need for a separation step as part of the assay, and the need for an absorbance spectrometer for quantification make the assay somewhat complicated and expensive. Future work will aim to move the assay toward a simple lateral flow assay format, which may be able to eliminate the separation step and the need for instrumentation to quantify the assay output. Even so, the current assay is suitable for high throughput analysis of patient eosinophilia without the need for measurement of cell differentials, which is a tedious and time-consuming practice. 

In summary, a highly specific and sensitive DNA aptamer for EPX was obtained from a magnetic bead-based SELEX method. A colorimetric aptamer-pulldown assay was developed which yielded a limit of detection of ~ 1 nM in buffer and in spiked sputum samples which is comparable to the relevant clinical values^10,25^. Our initial validation results of the aptamer-pulldown assay with clinical samples are promising. We believe that after further optimizations and validation with more clinical samples, this new assay will provide an inexpensive, quick, and accurate method to identify and monitor airway eosinophilia in patients with a variety of airway diseases, and to direct anti-inflammatory treatment including inhaled and oral corticosteroids and anti-Th2 biologics that primarily targets eosinophils.

## Methods

### Materials

#### Oligonucleotides and chemicals

All DNA oligonucleotides (Table S1) were obtained from Integrated DNA Technologies (Iowa, USA), and purified by standard 10% dPAGE (7 M urea) prior to use. NHS-Activated Magnetic beads (Prod. #88,826) and Pierce streptavidin-coated agarose beads (Prod. #20,349) were purchased from Thermo Scientific (Burlington, Ontario). Biotools DNA polymerase (#BTL-10043) was purchased from Mandel Scientific (Guelph, Ontario). Eosinophil peroxidase (EPX, #342-60-0.1), Myeloperoxidase (MPO, #426-10-0.1), Thyroid peroxidase (TPO, #ABIN934717) and Lactoperoxidase (LPO, #LS000151) were purchased from Cedarlane (Burlington, Ontario). Enhanced K-blue TMB substrate was from Neogen Corporation (#308,175, KY, USA). Buffers are outlined in Table S2. All other chemicals were purchased from Sigma-Aldrich and used without further purification.

#### Instruments

A Tecan M1000 plate reader was used for microplate fluorescence readings at an excitation wavelength of 490 nm and emission at 520 nm, with the exception of fluorescence anisotropy which was obtained using excitation at 470 nm and emission at 520 nm to reduce scattering artifacts. PCR was performed using a Dual 48 Bio-Rad thermal cycler. Agarose gels were visualized using a Bio-Rad ChemiDoc imaging system and quantified using Image Lab 6.0 software. Pictures of the microwell plates and microcentrifuge tubes used in the EPX pulldown assay were taken using a Samsung Galaxy S9 cellular phone operated in automatic mode and processed as noted below. Figures were made using GraphPad Prism 5 or Excel 2016 software.

#### Conjugating EPX/MPO to magnetic beads

Proteins were coupled to the magnetic beads (MBs) as per the manufacturer’s protocol. Briefly, 300 µL of MBs were mixed with Cwb A (see Table S2 in the SI) for 15 s, then placed in a magnetic stand to collect the beads and discard the supernatant. EPX (for positive selection) or MPO (for counter selection) was then added and mixed on a 360° microfuge tube rotator for two hours at room temperature. After brief centrifugation using benchtop centrifuge machine, the MBs were separated with a magnetic stand and the supernatant was saved for quantification of unbound protein via Bradford assay. The MBs were washed with 500 µL of Cwb B (See buffer composition in Table S2 in SI) for 15 s, then the supernatant was discarded. This washing with Cwb B was repeated two more times, followed by two washes with ddH_2_O. Qb was then added for two hours to quench any unreacted amine moieties, followed by three washes with ddH_2_O. The beads were suspended in Sb and kept at 4 °C until use.

#### In vitro aptamer selection

The selection protocol was adapted from two previous reported protocols and is outlined in Fig. 1B^31^. Both EPX- and MPO-coated MBs were washed 5 × using 1 × WB prior to use. One nanomole of DNA library (DNA_L_) was dissolved in 50 µL of 1 × SB, heated at 90 °C for 5 min then cooled at room temperature for 20 min. This solution was added to 500 µL of MPO-coated MBs suspended in 1 × SB, mixed and incubated at room temperature for 30 min with mild shaking. The MBs were separated by a magnetic separator and the unbound free DNA molecules in the clear supernatant were collected. These unbound DNA molecules in the supernatant were then added to the suspension of EPX-coated MBs for positive selection (500 µL volume, approximately 50 pmol of EPX) and incubated at room temperature with mild shaking for 2 h. Then, the MBs were separated with a magnetic separator and the supernatant was discarded. The MBs were washed 9 × using 1 × WB (500 µL each). After washing, the bound DNA molecules were eluted from the beads by heating the beads at 80 °C for 40 min in 300 µL elution buffer (EB: composition is shown in Table S2). The DNA molecules in EB was then precipitated by standard ethanol precipitation and resuspended in 50 µL of ddH_2_O. One microliter of this DNA solution was used in PCR amplification.

The PCR was conducted in 50 µL volume in two steps with two sets of primers called PCR1 and PCR2^32^. The PCR1 mixture contained 1 µL of the eluted DNA from the positive selection along with 1 µM FP1, 1 µM RP1, 200 mM dNTPs, 1 × PCR reaction buffer (75 mM Tris–HCl, 2 mM MgCl_2_, 50 mM KCl, 20 mM (NH_4_)_2_SO_4_, pH 9), and 2.5 U Biotools DNA polymerase. Thermal cycles were performed as follows: 95 °C for 60 s; ~ 15–18 cycles of 90 °C for 45 s, 53 °C for 45 s, 70 °C for 45 s and finally 5 min incubation at 70 °C for extension. PCR product was analyzed by agarose gel electrophoresis (2% w/v containing 1 × SYBR GOLD) to ensure sufficient PCR product. A portion of PCR1 product was diluted 20 × and 1 µL of this diluted PCR1 product was applied in PCR2 in 50 µL with the same condition of PCR1 except using FP2 and RP2 primer set (sequences are given in Figure Table S1). The triethylene glycol spacer in RP2 prevents the amplification of the poly-A region of RP2, resulting in the aptamer sequence being 20 nucleotides shorter than the antisense strand. The aptamer was purified using denaturing dPAGE and used in the next round of selection in the same way as described for the first round of selection. The selection and enrichment by PCR were iterated until round seven. In round seven, the DNA pool was divided into two. The same selection was continued with the first portion, while the second portion was applied in a modified selection protocol wherein the EPX-coupled MBs were blocked by adding 1 µM blocker DNA (Table S1) before adding the purified DNA_L_ of round 7. Both selections were continued till 15 rounds and the DNA population of each selection employed in deep sequencing (McMaster University, DNA sequencing facility). The amount of MBs and DNA pool of each round of selection can be found below in Table S3.

#### Electrophoretic mobility shift assay (EMSA)

The EPX binding of top 5 aptamers from each selection were analyzed by EMSA. Binding reactions were performed in 10 µL of 1 × SB containing 3 nM fluorescently labelled DNA, 1 ng poly(dI-dC) and target protein (concentrations ranging from 0 to 100 nM). After binding for 60 min, 10 µL of native loading buffer (1 × SB + 40% w/v sucrose) was added and the samples were loaded into a 0.3% w/v agarose gel. The gel was visualized and analyzed by Bio-Rad ChemidocTM imager.

#### Anisotropy of DNA/protein interactions

Fluorescence anisotropy was performed in 50 µL reactions containing 3 nM aptamer in buffer (PBS, 0.5 × SB or 1 × SB) and protein (EPX or MPO) ranging from 0 to 100 nM. A G-factor of 1.105 was determined by calibrating the fluorimeter with 1 nM fluorescein in 10 mM NaOH, and used to correct for the polarization bias of the system. All samples were prepared in duplicate, then measured in triplicate after reacting for 60 min at room temperature.

#### EPX pulldown assay

Biotinylated EAP1-05T3 was immobilized on streptavidin-coated agarose beads as follows: 200 µL of streptavidin-coated agarose slurry was transferred into a 1.5 mL Eppendorf tube and washed with 500 µL of 1 × HB (50 mM HEPES, 300 mM NaCl, 15 mM MgCl2, 0.01% Tween20, pH 7.5). 1 nmole of biotinylated EAP1-05T3 was added to this slurry and rotated gently for 1 h to bind the aptamers with the beads. The beads were washed 5 × with 500 µL of 1 × HB each time and finally suspended in 1000 µL of 1 × HB and stored at 4 °C until use. 50 µL of this bead slurry was used for each pulldown experiment.

#### Specificity test

50 µL of aptamer-conjugated beads from above step were aliquoted in each tube and labelled for each experiments: Buffer, EPX, MPO, LPO, TPO and BSA respectively. 1 µM stock of each of protein was made in 1 × HB. Next, 1.0 µL of each protein was added to their respective tube and incubated at room temperature for 30 min with occasional pipetting for homogeneous binding. Only buffer was added in the control experiment tube that was labelled as buffer. Then the beads were sedimented by brief centrifugation using a benchtop centrifuge and washed 3 × using 300 µL 1 × HB. Next, the beads were resuspended in 25 µL HB. 35 µL of TMB solution (Neogen’s Enhanced K-blue substrate) was added to each tube. After pipette mixing the tubes were kept at room temperature for color development. Color was captured using a smartphone camera at 5 min. Then, 70 µL of 0.5 M HCl was added to the tube to quench the peroxidase reaction. This yellow solution was immediately transferred into 96 well plate and the OD was measured at 450 nm using Tecan M200 plate reader. The data was processed using Microsoft excel software.

#### Sensitivity test in buffer

EPX stocks of different concentrations (10.0, 5.0, 2.5, 1.25, 0.625, 0.312, 0.156, 0.078, 0.039 and 0.019 µM) were made in 1 × HB. Similar to specificity test, 50 µL of aptamer conjugated beads slurry was transferred in each fresh Eppendorf tube (3 tubes for each concentration for triplicate experiments). 1.0 µL of each protein was added to their respective tube and incubated at room temperature for 30 min with occasional pipetting for homogeneous binding. Only buffer was added in the control experiment tube that was labelled as buffer. Then the beads were sedimented by brief centrifugation using a benchtop centrifuge and washed 3 × using 300 µL 1 × HB. Next, the beads were resuspended in 25 µL HB. 35 µL of TMB solution (Neogen’s Enhanced K-blue substrate) was added to each tube. After pipette mixing the tubes were kept at room temperature for color development. Color was captured by smartphone camera at 5 min. Then, 70 µL of 0.5 M HCl was added to the tube to quench the peroxidase reaction. This yellow solution was immediately transferred into 96 well plate and the OD was measured at 450 nm using Tecan M200 plate reader. The data was processed using Microsoft Excel 2016 software.

#### Sensitivity test in sputum

Similar to the sensitivity test in buffer in the above step, EPX stocks of different concentrations (10.0, 5.0, 2.5, 1.25, 0.625, 0.312, 0.156, 0.078, 0.039 and 0.019 µM) were made in in HB. The beads were suspended in HB including 20% sputum sample. 50 µL of aptamer conjugated beads slurry was transferred in each fresh Eppendorf tube (3 tubes for each concentration for triplicate experiments). 1.0 µL of protein from each stock was added to their respective tube and incubated at room temperature for 30 min with occasional pipetting for homogeneous binding. Only buffer was added in the control experiment tube that was labelled as buffer. Then the beads were sedimented by brief centrifugation using a benchtop centrifuge and washed 3 × using 300 µL 1 × HB. Next, the beads were resuspended in 25 µL HB. 35 µL of TMB solution (Neogen’s Enhanced K-blue substrate) was added to each tube. After pipette mixing the tubes were kept at room temperature for color development. Color was captured by smart cell phone at 5 min. Then, 70 µL of 0.5 M HCl was added to the tube to quench the peroxidase reaction. This yellow solution was immediately transferred into 96 well plate and the OD was measured at 450 nm using Tecan M200 plate reader. The data was processed using Microsoft Excel 2016 software.

#### EPX pulldown assay of the clinical samples

##### Sample processing (old method; see Fig. S6^24^)

All experiments with patient samples were performed as per the protocols approved by the Hamilton integrated Research Ethics Board (HiREB), St. Joseph’s Hospital, Hamilton, project number:12-3687. Healthy donors were recruited under the approved HiREB protocol #13,203 and signed written consent was obtained for this clinical validation study. All methods were performed in accordance in compliance with relevant guidelines and regulations. The collected sputum was transferred in a fresh petri dish by pipette and put under an inverted microscope. The sputum was selected based on the cell morphology and separated. This desired sputum was then transferred in a pre-weighed 15 mL conical tube and the total amount of sputum was calculated by deducting the tube weight. Next, the sputum was dispersed by adding 8 × of the PBS (137 mM NaCl, 2.7 mM KCl. 10 mM Na_2_HPO_4_, 1.8 mM KH_2_PO_4_, pH 7.4 including 4 mM DTT) and gently inverting the tube. The samples were then aliquoted 250 µL in fresh Eppendorf tubes and stored at – 20 °C until use.

##### Sample processing (improved method, see Fig. S6)

Sputum samples were collected with the consent of each patient. The sputum of each person was first put in a petri dish on a black background (using a black piece of paper) to visualise the opaque sputum plugs. If the sputa expectorate was too thick, forceps was used to select the sputum plugs without the help of a microscope. The sputum plugs were then transferred to a pre-weighed fresh conical 15 mL tube. The amount of sputum plug (weight) was calculated by deducting the tube weight. Next, the sputum was dispersed by adding 8x (weight) of the HB (50 mM HEPES, 300 mM NaCl, 15 mM MgCl_2_, 0.01% Tween20 pH 7.5 including 2 mM DTT) and gently inverting the tube. The samples were dispersed by inverting the tube by hand for 5 min and then settling the dispersal on ice for 2 min. The supernatants from the settled dispersed samples were then aliquoted into 250 µL in fresh Eppendorf tubes that included a protease inhibitor cocktail (Roche catalogue number 11697498001, containing a mixture of serine, cysteine and metalloproteases—1 tablet is dissolved in 50 mL deionized water as per manufacturer’s protocol and 10 µL is added to 500 µL of dispersed supernatants) and stored at − 20 °C until use. All reported sputa were collected using this method in addition to the above routine method.

#### Aptamer pull-down assay

The stock of aptamer agarose beads was prepared in the same way as described above. 50 µL of the aptamer-bead conjugate was transferred to a fresh microcentrifuge tube. 50 µL of the above processed sample was added to this tube and mixed. The tube was rotated vertically to prevent the beads from settling down. After 30 min of incubation, the tube was briefly centrifuged to separate the beads. The supernatant was carefully discarded. The beads were washed three times (300 µL in each wash) with HB. The beads were then suspended in 25 µL of HB. This was followed by the addition of 35 µL of TMB solution (Neogne’s ready-to-use K-blue ready substrate) and allowed to develop color. For all tube-based assays in Figs. 4 and 5, a photograph of the tube was obtained after a 5 min reaction time using a Samsung galaxy S9 smartphone camera from a constant height, with the flash off. The images were saved as JPEG files and processed using ImageJ software. The contrast and brightness was adjusted keeping the brightness and contrast the same for all images (brightness: 50, contrast: 75). Composite images were generated by cropping and then combining individual images of reaction tubes into a single image using Microsoft PowerPoint 2016, with final images exported as TIFF images. To obtain quantitative absorbance readings, 70 µL of 0.5 M HCl was added to each tube to quench the peroxidase reaction. This yellow solution was immediately transferred to 96 well plate and the OD was measured at 450 nm using Tecan M200 plate reader. The data was processed using Microsoft Excel 2016 software. The aptamer pulldown assays were conducted identically with all reported samples (healthy and patient-derived).

## Supplementary Information

Supplementary Information.

## Data Availability

High throughput sequencing data for the pool obtained in selection round 15 can be accessed in the NCBI's Gene Expression Omnibus (GEO) public archive using accession code GSE213923 at the following link: https://www.ncbi.nlm.nih.gov/geo/query/acc.cgi?acc=GSE213923.
