# Supplementary material for: A sputum bioassay for airway eosinophilia using an eosinophil peroxidase aptamer
Source: Sci Rep. 2022 Dec 28;12:22476. doi: 10.1038/s41598-022-26949-7 (PMC9797489; doi:10.1038/s41598-022-26949-7)
Supplement: Supplementary file 1 — Supplementary Information. [file 41598_2022_26949_MOESM1_ESM.pdf]

A sputum bioassay for airway eosinophilia using an eosinophil peroxidase aptamer

M. Monsur Ali,[†§] Michael G. Wolfe,[†§] Manali Mukherjee,[ⱡ §] Katherine Radford,[ⱡ] Zil Patel,[ⱡ] Dawn White,[†] Julijana Milojevic,[†] Alfredo Capretta,[†] Parameswaran Nair*,[ⱡ] and John D. Brennan*[†]

[†] Biointerfaces Institute, McMaster University, 1280 Main Street West, Hamilton, ON, L8S 4O3 (Canada).

[ⱡ] Division of Respirology, McMaster University, and Firestone Institute of Respiratory Health at St. Joseph's Health Care, Hamilton ON L8N 4A6 (Canada)

[§]These authors contributed equally to this work.

*Correspondence: brennanj@mcmaster.ca, parames@mcmaster.ca

Table S1: Sequences used in the study

Name	Description	Sequence
DNA_L	DNA Library	ATGCCATCCTACCAACN_{40}GAGCTCTGAACTGG
FP1	Forward primer 1	ATGCCATCCTACCAAC
FP2	Forward primer 2	/56-FAM/ATGCCATCCTACCAAC
RP1	Reverse primer 1	CCAGTTCAGAGCTC
RP2	Reverse primer 2	AAAAAAAAAAAAAAAAAAAA/isp9/CCAGTTCAGAGCTC
DNA_B	Blocker DNA	GCGGCCCATTCTTCATTTTAGGGCCGC
EAP1-05T1FAM	Truncated EAP1-05	/56-FAM/CCATCCTACCAACCAGGGGGACAGTGCAAAGGGGTAGGGAG
EAP1-05T2FAM	Truncated EAP1-05	/56-FAM/ATGCCATCCTACCAATAGGGAGGGGGCTAGGGGGGAGCTCTGAACTCG
EAP1-05T3FAM	Truncated EAP1-05	/56-FAM/ATGCCATCCTACCAACCAGGGGGACAGTGCAAAGGGAGCTCTGAACTCG
EAP1-05T4FAM	Mutated EAP1-05	/56-FAM/ATGCCATCCTACCAACCAGAAAGACAGTGCAAAGAAATAGAAAGAGAGCTAGAAGAAAGCTCTGAACTCG
EAP1-05T3BT	Biotinylated EAP1-05T3	/5Biosg/TTTTTTTTTTATGCCATCCTACCAACCAGGGGGACAGTGCAAAGGGAGCTCTGAACTCG

Table S2: Buffers used in the study

Name	Description	Contents
1× SB	Selection buffer	50 mM Tris, 1 M NaCl, 5 mM $MgCl_2$, 2 mM KCl, 0.01 % v/v Tween 20, pH 9
1× WB	Wash buffer	50 mM Tris, 1 M NaCl, 5 mM $MgCl_2$, 2 mM KCl, 0.1 % v/v Tween 20, pH 9
EB	Elution buffer	50 mM Tris, 4 M guanidine thiocyanate, 1 mM DTT, pH 9
Cwb A	Coupling wash buffer A	1 mM Ice-cold HCl
Cwb B	Coupling wash buffer B	0.1 M glycine, pH 2
Cb	Coupling buffer	50 mM sodium tetraborate, pH 8.5
Qb	Quenching buffer	3 M ethanolamine, pH 9
Sb	Storage buffer	Coupling buffer + 0.05% w/v NaN_3
PBS	Phosphate buffer saline	137 mM NaCl, 2.7 mM KCl. 10 mM Na_2HPO4, 1.8 mM KH_2PO4, pH 7.4
HB	HEPES buffer	50 mM HEPES, 150 mM NaCl, 15 mM $MgCl_2$, 0.01% Tween20, pH 7.5

Table S3: SELEX outline for the EAPT1 and EAPT2 selections including times and values used. T1 selection was performed without the blocker oligo, while the T2 selection was performed with the blocker oligo.

**Round**	**DNA library (pmol)**	**Beads**	**Positive selection time (minutes)**	**Negative selection (minutes)**	**Blocker DNA, pmol (blocker:target)**
**1**	1000	1×10^7 beads	120	30	500 (10:1)
**2**	~ 50	1×10^6 beads	60		50 (10:1)
**3**		1×10^6 beads	60	30	50 (10:1)
**4**		1×10^6 beads	30		100 (20:1)
**5**		1×10^5 beads	30	30	10 (20:1)
**6**		1×10^5 beads	15		10 (20:1)
**7**		1×10^5 beads	15	30	20 (40:1)
**8**		1×10^4 beads	15		2 (40:1)
**9**		1×10^4 beads	15	30	2 (40:1)
**10**		1×10^4 beads	10		2 (40:1)
**11**		1×10^4 beads	10	30	4 (80:1)
**12**		1×10^4 beads	10		4 (80:1)
**13**		1×10^4 beads	5	30	8 (160:1)
**14**		1×10^4 beads	5		8 (160:1)
**15**		1×10^4 beads	5	30	8 (160:1)

Table S4: Airway inflammatory status for individual patients based on sputum cytology.

Patient Number	Intact Sputum Eosinophils (%)	Free Eosinophil Granules (FEGs)	Inflammatory phenotype	Inflammatory status (Eos. = 1, Non-eos. = 0)	OD from aptamer pulldown assay (cut-off, 0.32)	Agreement with gold standard (yes = 1)
**Healthy Controls**						
H1	0	0	Pauci	0	0.1202	1
H2	0	0	Pauci	0	0.0969	1
H3	0	0	Pauci	0	0.1393	1
H4	0	0	Pauci	0	0.1605	1
H5	0	0	Pauci	0	0.0989	1
H6	0.5	0	Pauci	0	0.0930	1
H7	0	0	Pauci	0	0.2974	1
H8	0.3	0	Pauci	0	0.0936	1
H9	1.3	0	Pauci	0	0.4603	0
H10	0	0	Pauci	0	0.1432	1
**Clinically-indicated Patients**						
P1	0	0	Trivial Neutrophilic	0	0.1436	1
P2	0	0	Pauci	0	0.2085	1
P3	0.7	0	Pauci	0	0.2402	1
P4	0	0	Infective Neutrophilic	0	0.2281	1
P5	0	0	Pauci	0	0.0968	1
P6	0.5	0	Infective Neutrophilic	0	0.1114	1
P7	0.3	0	Pauci	0	0.1829	1
P8	DG*	0	Pauci	0	0.0939	1
P9	0.5	0	Infective Neutrophilic	0	0.0976	1
P10	0	0	Pauci	0	0.1042	1
P11	0.8	1	Pauci	1	0.1088	0
P12	0.3	0	Pauci	0	0.3127	1
P13	0	0	Pauci	0	0.1090	1
P14	0	0	Infective Neutrophilic	0	0.2298	1
P15	0	0	Pauci	0	0.1790	1
P16	0.3	0	Infective Neutrophilic	0	0.2109	1
P17	0.8	1	Pauci	1	0.1030	1
P18	0	0	Infective Neutrophilic	0	0.1558	1

P19	0	0	Infective Neutrophilic	0	1.4479	0
P20	39.8	3	Eosinophilic	1	1.0566	1
P21	0.5	0	Infective Neutrophilic	0	0.8023	0
P22	0.3	0	Infective Neutrophilic	0	0.3890	0
P23	DG*	3	Eosinophilic	1	1.0331	1
P24	17.8	1	Mixed	1	0.6264	1
P25	1	1	Mixed	1	0.8018	1
P26	12.1	3	Eosinophilic	1	0.4084	1
P27	8	3	Eosinophilic	1	0.3573	1
P28	DG*	3	Eosinophilic	1	0.7481	1
P29	13.3	3	Mixed	1	1.1791	1
P30	37.5	3	Eosinophilic	1	1.1612	1
P31	DG*	3	Eosinophilic	1	1.0408	1
P32	DG*	1	Pauci	1	0.2639	1
P33	3.5	0	Eosinophilic	1	0.7202	1
P34	8.5	3	Eosinophilic	1	1.2495	1
P35	70	3	Eosinophilic	1	0.4366	1
P36	4.3	1	Eosinophilic	1	0.1157	0

NB - all inflammatory phenotypes have been assigned based on routine sputum cytology from Belda et al., AJRCCM 2000.
Pauci – pauci granulocytic, Mixed – mixed granulocytic.
P – clinically-indicated patient sputum, H - healthy control sputum
*DG – indicates cell counts that were unreliable due to intense degranulation

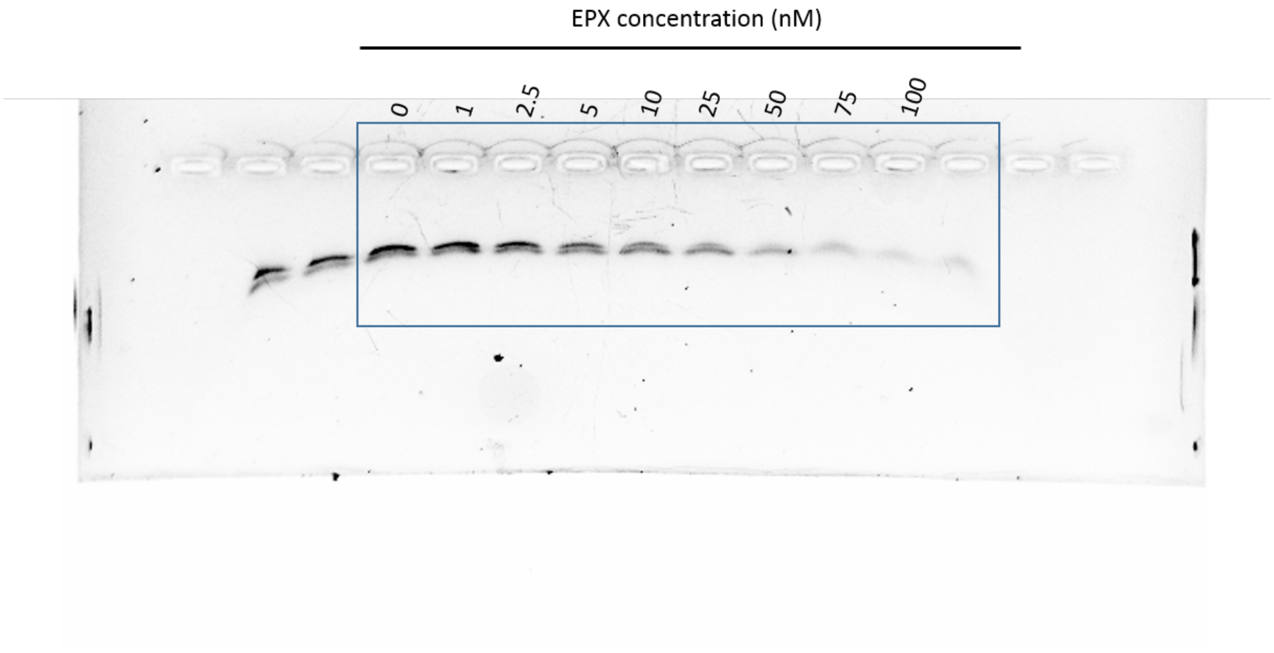

Figure S1: Unprocessed gel image (boxed) used in Figure 1E.

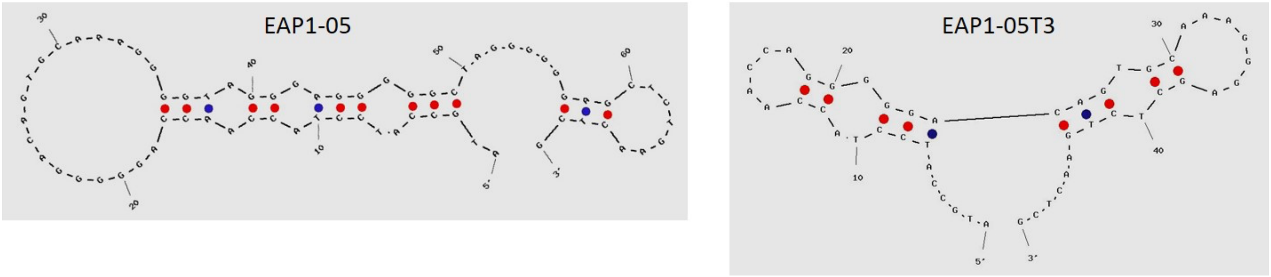

Figure S2: Predicted secondary structures of EAP1-05 and EAP1-05T3

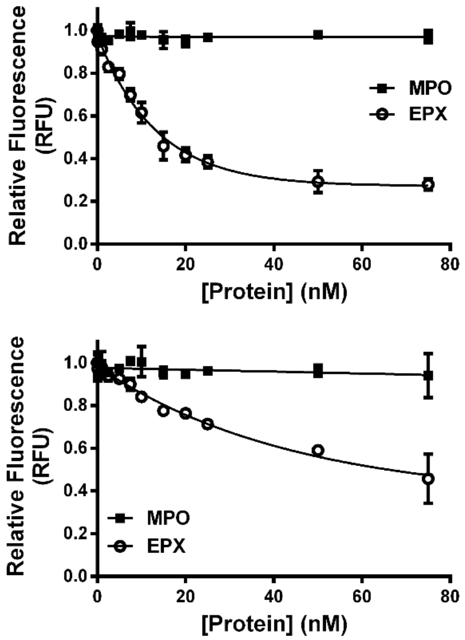

Figure S3: Fluorescence quenching of EPX upon binding with aptamer EAP1-05 and EAP1-05T3.

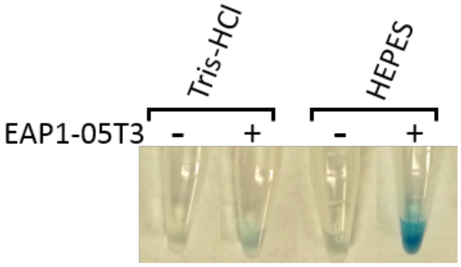

Figure S4: Effect of buffer on the pulldown assay.

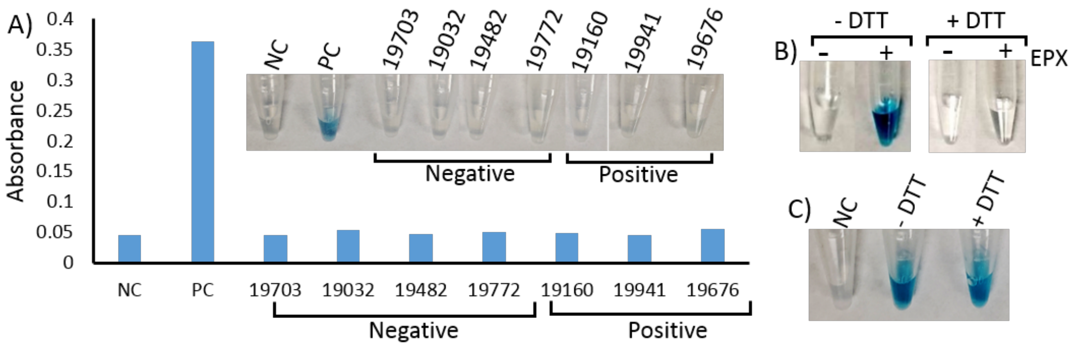

Figure S5: Effect of PBS and DTT in the pulldown assay.

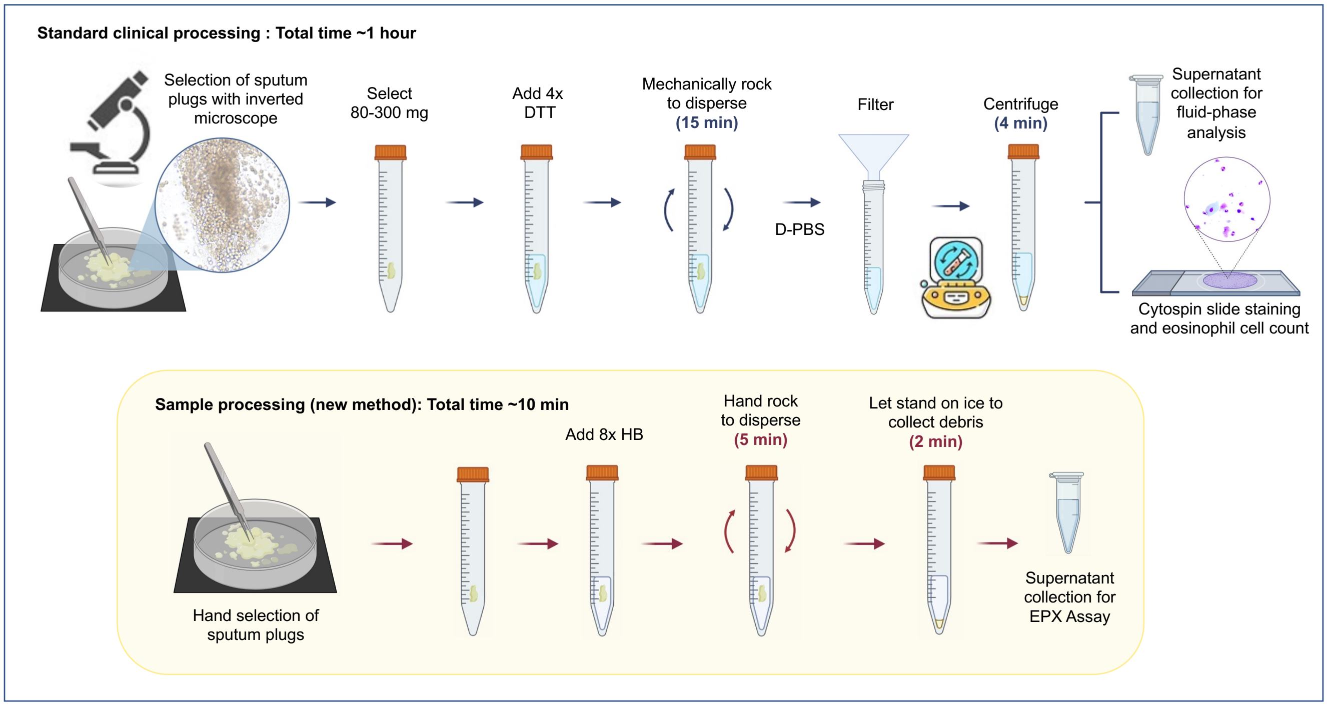

Figure S6. Comparison of sputum sample processing steps for the original method (top) and the new method (bottom), showing the reduction in the number of steps and technical complexity for the new sample processing method.

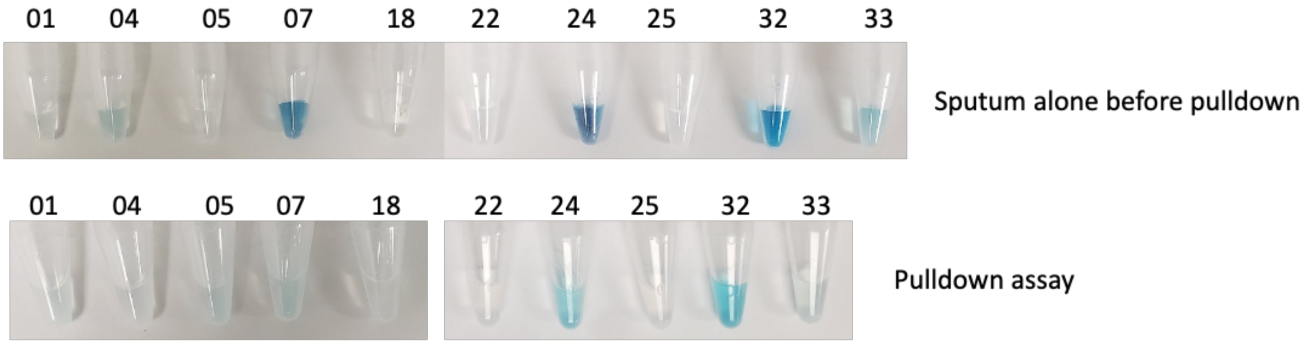

Figure S7. Visual output of peroxide/TMB assay for processed sputum prior to performing the aptamer pull-down assay on 10 healthy sputum samples.

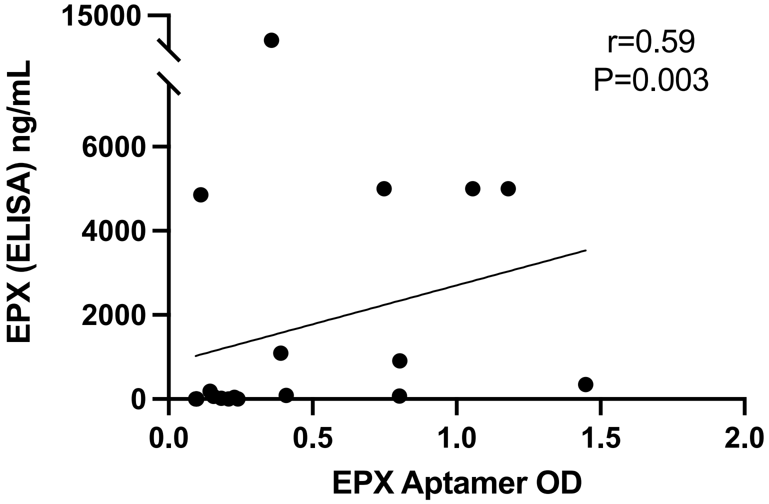

Figure S8. Correlation plot for EPX Aptamer values with EPX ELISA in HEPES buffer-dispersed sputum samples
